# Effects of Chlorich^®^EnergyBoost on Enhancing Physical Performance and Anti-Fatigue Properties in Mice

**DOI:** 10.3390/foods13142232

**Published:** 2024-07-16

**Authors:** Shih-An Yang, Po-Hsun Cheng, Yi-Ju Hsu, Shu-Feng Cheng, Meng-Hsueh Amanda Lin, Chi-Chang Huang

**Affiliations:** 1 Product Development & Research Institute, Vedan Biotechnology, Taichung 43351, Taiwan; yang63@mail.vedan.com (S.-A.Y.); paul@mail.vedan.com (P.-H.C.); shufeng@mail.vedan.com (S.-F.C.); 2 Graduate Institute of Sports Science, National Taiwan Sport University, Taoyuan 33301, Taiwan; ruby780202@ntsu.edu.tw

**Keywords:** *Chlorella sorokiniana*, exercise performance, anti-fatigue, energy boost, muscle strength, chlorich, energy, VB-1974, polysaccharide

## Abstract

Chlorich^®^EnergyBoost, a water extract obtained from *Chlorella sorokiniana*, has been proposed to enhance physical performance and provide anti-fatigue effects. This study assessed the impact of Chlorich^®^EnergyBoost supplementation on physical performance and its anti-fatigue properties. Twenty-four mice were allocated into four groups: (1) the control group receiving only water,;(2) the 1X group (49.2 mg/kg/day); (3) the 2X group (98.4 g/kg/day); and (4) the 5X group (246 g/kg/day). All groups were orally administered the supplements for four consecutive weeks. The evaluation included grip strength, swimming endurance, an exhaustion test, and serum biochemistry analysis. Additionally, the study examined the bioactive peptides through matrix-assisted laser desorption/ionization mass spectrometry (MALDI-TOF MS) and conducted bacterial reverse mutation and acute oral toxicity tests for safety assessment. The findings indicated that Chlorich^®^EnergyBoost supplementation led to a significant reduction in serum lactate levels by 14.08% to 22.54% and blood urea nitrogen levels by 12.23% to 16.76%, an increase in the lactate clearance rate by 0.28 to 0.35, an enhancement of muscle glycogen storage by 1.10 to 1.44-fold, and hepatic glycogen storage by 1.41 to 1.47-fold. These results demonstrated dose-dependent effects. MALDI-TOF analysis revealed the expression of dihydrolipoamide dehydrogenase and superoxide dismutase. Both the bacterial reverse mutation and acute oral toxicity tests showed no adverse effects.

## 1. Introduction

Physical inactivity stands as a prominent risk factor for global mortality [1]. As per the World Health Organization (WHO), “Almost 500 million people will develop heart disease, obesity, diabetes or other noncommunicable diseases (NCDs) attributable to physical inactivity, between 2020 and 2030, costing US$ 27 billion annually, if governments don’t take urgent action to encourage more physical activity among their populations” [2]. The link between NCDs and physical inactivity has been established, with a sedentary lifestyle (such as prolonged sitting, driving, watching television, or computer use) leading to metabolic dysfunction, changes in cardiac output, muscle composition, and an increase in waist circumference [3,4,5]. Research has shown that a 10% increase in sedentary time results in a 3.1 cm rise in waist circumference, correlating with metabolic risks, being overweight, and obesity [4]. Additionally, another study indicated a 20% increase in overall cancer risk due to physical inactivity [6]. The WHO projects approximately 499 million new cases of NCDs by 2030, with an associated health care cost estimated at 520 billion international dollars (INT) [4,5,6]. However, it is worth noting that physical activity can trigger a predictable stress response in the body, leading to fatigue and compromised immunity. Feelings of fatigue and weakness serve as primary obstacles hindering individuals from participating in physical activities [7,8,9,10,11].

One common constraint in daily activities is fatigue, particularly among individuals with inadequate muscle strength [12]. Fatigue is characterized by a lack of energy and/or feelings of tiredness. The development of fatigue and the decrease in energy levels are commonly observed not only in adults but also in the elderly population [11,12,13]. With the expanding global population and changing lifestyles, traditional nutrient-dense foods are increasingly insufficient to fulfill the nutritional requirements of individuals. Consequently, there is a rising interest in and necessity for natural nutraceutical supplements that enhance physical activity, boost energy levels, alleviate fatigue, and enhance muscle strength [14]. Among these, *chlorella* has emerged as a viable nutraceutical ingredient that promotes physical strength and reduces fatigue, attributed to its bioactive compounds such as microalgae polysaccharides (MLP) [15,16,17,18].

*Chlorella*, a genus of freshwater single-celled green algae, is highly regarded for its extensive nutraceutical benefits to human health. Both animal and human studies have demonstrated its health-promoting effects, including immune system modulation, blood sugar regulation, enhanced physical activity, and muscle strength [19,20,21]. Previous research has also indicated that *chlorella* improves exercise performance by up-regulating monocarboxylate transporters and enhancing the activity of enzymes such as lactic dehydrogenase, citrate synthase, and cytochrome oxidase [15,22,23]. Specific bioactive compounds found in *chlorella*, such as polysaccharides, polyphenols, and peptides, have shown significant potential for enhancing physical performance and reducing fatigue. Furthermore, several toxicology studies have shown no toxicity or adverse effects in *chlorella* extract using the Sprague Dawley (SD) rat model [24,25].

*Chlorella sorokiniana* emerges as a prime candidate for the extraction of essential nutraceuticals, notably polysaccharides and peptides, ideal for a diverse range of health-related applications [15,18,26,27]. Consequently, *Chlorella sorokiniana* distinguishes itself as a highly favorable source for isolating nutraceutical compounds, specifically polysaccharides and peptides, renowned for their health-promoting properties and enhancement of physical strength [18,27]. Hence, it is recommended that *Chlorella sorokiniana* be recognized as a preeminent strain for the extraction of nutraceutical compounds, particularly polysaccharides tailored for nutraceutical applications.

In this investigation, Chlorich^®^EnergyBoost was extracted utilizing an enzymatic approach. The primary aim of this research is to evaluate the influence of supplementing with *Chlorella sorokiniana* extract on augmenting physical strength and its anti-fatigue properties. The hypothesis posits that Chlorich^®^EnergyBoost enhances physical strength and mitigates fatigue by facilitating rapid lactate clearance and minimizing muscle depletion. Furthermore, the study assessed the acute toxicity implications of Chlorich^®^EnergyBoost. The bioactive composition of Chlorich^®^EnergyBoost is detailed in Table S1.

## 2. Materials and Methods

### 2.1. Materials

The investigational product, Chlorich^®^EnergyBoost, is derived from *Chlorella sorokiniana* VB-1974, formerly identified as *Chlorella pyrenoidosa* [20,26]. It is meticulously crafted using an enzymatic extraction method to safeguard the integrity of its bioactive constituents, encompassing polysaccharides, peptides, and amino acids. Our analysis, although unpublished, identifies MLP as the principal bioactive compound within Chlorich^®^EnergyBoost. A detailed breakdown of the nutraceutical composition present in Chlorich^®^EnergyBoost is outlined in Table S1.

### 2.2. Matrix-Assisted Laser Desorption/Ionization Time-of-Flight Mass Spectrometry

MALDI-TOF MS was employed to analyze the peptide structure in Chlorich^®^EnergyBoost. The sample was prepared with a matrix substance, deposited on a MALDI plate, and subjected to laser ionization for precise peptide profiling [26,28,29].

### 2.3. Bacterial Reverse Mutation Test

The genotoxic potential of Chlorich^®^EnergyBoost was assessed using the Ames test with *Salmonella typhimurium* strains. This assessment adhered to the guidelines set forth by the Organisation for Economic Cooperation and Development (OECD) for chemical testing (Test No. 47, 2020) [30], as well as the protocols stipulated by the Ministry of Health and Welfare, Taiwan, R.O.C., for safety evaluations of health food (2020) [31]. Initial dose range finding determined 5 mg/plate as the highest feasible dose, followed by four descending concentrations across each strain for a comprehensive evaluation of genotoxic effects.

### 2.4. Acute Oral Toxicity Test

Twenty SD rats, evenly distributed across genders, were utilized in the acute oral toxicity evaluation. Chlorich^®^EnergyBoost was orally administered at a dose of 10,000 mg/kg on Day 1. The dosing regimen was repeated thrice at intervals of approximately 4–6 h within a 24-h timeframe, with water serving as the dosing vehicle at a volume of 10 mL/kg.

### 2.5. Animals and Experimental Design

The recommended daily dosage of Chlorich^®^EnergyBoost (Vedan Biotechnology, Inc., Taichung City, Taiwan) for an adult is 240 mg, calculated based on an average adult weight of 60 kg, equivalent to 4 mg per kg of body weight per day [32]. The daily recommended dosage of Chlorich^®^EnergyBoost ranged between 240 mg and 480 mg (two fold) for general public use and for optimal effects, respectively [31]. This dosage recommendation is tailored to achieve optimal therapeutic effects while adhering to safety thresholds [31]. To address metabolic rate variations between mice and humans, a conversion factor of 12.3, aligned with guidelines from the US Food and Drug Administration (FDA) and supported by prior studies, was applied [32,33]. For experimental purposes, the mouse equivalent dose was determined to be 49.2 mg/kg per day.

The study encompassed 24 adult Institute Cancer Research (ICR) mice, aged 6 weeks, with an average weight of approximately 32 g each. The mice were housed at the Animal Experiments Center within the Institute of Sports Science at the National Taiwan University of Sport. A two-week acclimation period preceded the experiment to facilitate the mice’s adjustment to the new environment, ensuring unrestricted access to food and water. Stringent environmental controls were maintained, including a temperature of 24 ± 2 °C, humidity at 65 ± 5%, and a consistent 12-h light-dark cycle, in compliance with guidelines established by the Institutional Animal Care and Use Committee (IACUC) [34] (approval number IACUC-10903, bioethical committee: Institute of Sports Science at the National Taiwan University of Sport IACUC). Following acclimation, the mice were randomly assigned to four groups, each comprising six animals: a control group (vehicle), a 1X dose group (one fold, 49.2 mg/kg), a 2X dose group (two fold, 98.4 mg/kg), and a 5X dose group (five fold, 246 mg/kg) of Chlorich^®^EnergyBoost. This stratification aimed to investigate the dose-dependent impacts of Chlorich^®^EnergyBoost. Throughout the experiment period, parameters such as the mice’s weight, food consumption, and water intake were meticulously monitored. Weight fluctuations were recorded from the initial to the final measurements to assess the health and growth effects of the varying dosages. A detailed timeline of the animal testing procedures is depicted in Figure S1.

### 2.6. Forelimb Grip Strength Assessment

In order to assess the impact of Chlorich^®^EnergyBoost on muscle strength, a digital conventional forelimb grip strength evaluation was conducted following four weeks of supplementation. This specialized test was designed to precisely measure the grip strength of the mice and was executed 30 min post feeding on the 29th day of the experimental period [35]. Details of the testing timeline are displayed in Figure S1.

### 2.7. Swimming Exercise Endurance Test

The influence of Chlorich^®^EnergyBoost on endurance performance was evaluated through a weight-loaded swimming test [36] on the 31st day of the experiment. Details of the testing timeline are displayed in Figure S1. Mice, after a 12-h fast, were given either the control solution or the supplement. Each mouse was equipped with a lead sheath on its tail, weighing 5% of its body weight, to provide resistance during swimming. Endurance was measured by the duration until exhaustion, defined as submersion without resurfacing for breathing within an 8-s interval.

### 2.8. Serum Biochemistry Analysis

Serum lactate, hepatic and muscular glycogen content, and blood urea nitrogen (BUN) levels were analyzed to assess the impact of Chlorich^®^EnergyBoost on fatigue-related biomarkers and physiological status. Blood samples were collected at different time points to monitor changes in lactate levels post exercise and evaluate muscle damage prevention [37]. Serum samples were processed using an automated analyzer (Hitachi 7060, Tokyo, Japan) for accurate quantification of biochemical markers.

### 2.9. Evaluation of Hepatic and Muscular Glycogen Content

Hepatic and muscular glycogen content were quantified on the 37th day of the study. Details of the testing timeline are displayed in Figure S1. Glycogen concentrations were determined in accordance with established protocols [37]. Tissues were postprandially collected, processed, and cryopreserved for glycogen quantification. Tissues were homogenized using a Bullet Blender Homogenizer (Next Advance, Cambridge, MA, USA). Commercial standards were used for calibration, and tissues were homogenized for analysis [37].

### 2.10. Statistic Analysis

Statistical analyses were performed utilizing the Statistical Analysis System (SAS). To discern differences among the experimental groups, one-way ANOVA was applied, followed by Duncan’s new multiple range test. Statistical significance was determined at *p* < 0.05. All outcomes are presented as mean ± standard deviation (SD).

## 3. Results

### 3.1. MALDI-TOF Mass Spectrometry Analysis

Within this investigation, MALDI-TOF MS was utilized for the compositional and structural analyses of Chlorich^®^EnergyBoost, specifically focusing on the mass-to-charge ratio (*m*/*z*) of sample ions [28,29]. The outcomes unveiled the presence of two notable enzymes in Chlorich^®^EnergyBoost. Dihydrolipoamide dehydrogenase was identified with a molecular mass of 82,458 Daltons, a score of 6204, an exponentially modified Protein Abundance Index (emPAI) of 0.87, 188 matches to the protein database, and a sequence coverage length of 15 amino acids (indicating identity). Similarly, superoxide dismutase was identified, exhibiting a molecular mass of 42,219 Daltons, a score of 351, an emPAI of 0.43, 13 matches, and a sequence coverage length of 4 amino acids. The analysis also emphasized specific peptides derived from these proteins. For dihydrolipoamide dehydrogenase, identified peptides encompassed sequences such as R.GATFLDPK.G, K.GQAVFSIAK.V, and R.VPFLFSVK.E. In the instance of superoxide dismutases (SODs), peptides like K.FVEAFK.A, K.LSIEEVMLK.T, and R.DLGGYDKFVEAFK.A were recognized, offering further insights into the protein composition of Chlorich^®^EnergyBoost.

### 3.2. Bacterial Reverse Mutation Test Demonstrating No Cytotoxic Effects

The bacterial reverse mutation test was executed to assess the potential cytotoxic effects of Chlorich^®^EnergyBoost. The results indicated no significant elevation in the number of revertant colonies for any of the tested concentrations of Chlorich^®^EnergyBoost, compared to the negative control, across all five tested strains of *Salmonella typhimurium*, both with and without the S9 metabolic activation mixture. The findings validate that Chlorich^®^EnergyBoost does not manifest cytotoxicity under the tested conditions, as depicted in Table S2.

### 3.3. Acute Oral Toxicity Assessment Indicating No Observable Effects

Throughout the acute oral toxicity study, no mortalities or test article-related clinical signs were observed among the SD rats. The results illustrate that Chlorich^®^EnergyBoost did not demonstrate any apparent acute toxicity effects following the administration of a 10,000 mg/kg dose. The study assessed body weight gains throughout the experiment, revealing no significant toxicity observations (Table S3). Both male and female groups sustained consistent weight gain without any abnormalities associated with the test article. Gross necropsy examinations (Tables S4 and S5) disclosed no test article-related alterations in any of the animals. This evaluation further substantiates the absence of acute toxic effects from Chlorich^®^EnergyBoost supplementation.

### 3.4. General Characteristics of Mice after Four Weeks of Chlorich^®^EnergyBoost Supplementation

Throughout the intervention period, the mice in all of the experimental groups displayed a consistent increase in body weight without notable variations between the groups. Additionally, there were no remarkable alterations observed in the dietary or water consumption across the diverse groups, indicating that the incremental doses of Chlorich^®^EnergyBoost did not have a significant impact on the feeding behaviors of the mice (refer to Table S6).

### 3.5. Effect of Chlorich^®^EnergyBoost Supplementation on Muscle Strength

**Figure 1:**
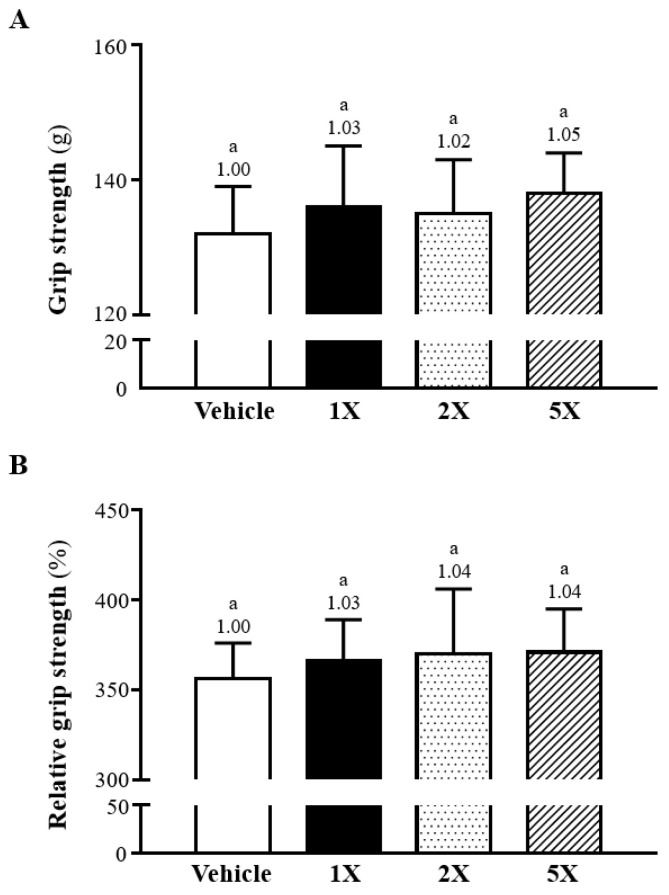
Effects of Chlorich^®^EnergyBoost supplementation on (**A**) Forelimb Grip Strength and (**B**) Relative Forelimb Grip Strength (%), calculated as a percentage of body weight. The supplementation levels for the Chlorich^®^EnergyBoost groups are denoted as 1X (One fold), 2X (Two fold), and 5X (Five fold), with the vehicle group serving as the control. All results are presented as mean ± SD (*n* = 6 mice per group). The presence of distinct superscript letters above each bar indicates statistically significant differences between the experimental groups (*p* < 0.05).

The impact of Chlorich^®^EnergyBoost on muscle strength was assessed by evaluating the forelimb grip strength following a four-week supplementation period. The measurements of grip strength for the vehicle control and the Chlorich^®^EnergyBoost supplemented groups (1X, 2X, and 5X) were recorded as follows: 132 ± 7, 136 ± 9, 135 ± 9, and 138 ± 6 (g), respectively (see Figure 1A). The analysis indicated no significant disparities in grip strength among the four groups. Considering that grip strength may vary with body weight, the relative grip strength, expressed as a percentage of body weight, was calculated to offer a standardized evaluation. The relative grip strengths for the Vehicle, 1X, 2X, and 5X groups were 356 ± 20, 366 ± 23, 370 ±36, and 371 ± 24 (%), respectively (see Figure 1B). These findings demonstrated no significant variances among the groups.

### 3.6. Enhancement of Exercise Performance by Chlorich^®^EnergyBoost Supplementation

**Figure 2:**
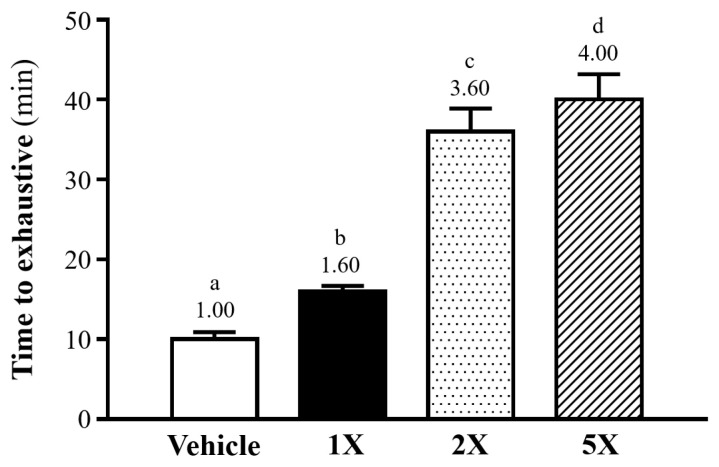
Effect of Chlorich^®^EnergyBoost Supplementation on Exhaustive Swimming Duration in Mice. The figure illustrates the significant extension in swimming durations across different supplementation levels: 1X (One fold), 2X (Two fold), and 5X (Five fold), with the vehicle group serving as the control. Each bar in the figure represents the mean ± SD (*n* = 6 mice per group), with different superscript letters (a, b, c, d) indicating statistically significant differences among the groups (*p* < 0.05).

The outcomes of the exhaustive swimming test, illustrated in Figure 2, exhibited notable enhancements in exercise performance within the Chlorich^®^EnergyBoost supplemented groups compared to the vehicle group. Specifically, the recorded swimming durations were 10.0 ± 0.9 min for the vehicle group, 16.0 ± 0.7 min for 1X, 36.0 ± 2.9 min for 2X, and 40.0 ± 3.2 min for 5X. The observed performance improvements were substantial, with 1X, 2X, and 5X showcasing exercise capacities 1.6 times *(p* = 0.0002), 3.6 times (*p* < 0.001), and 4.0 times *(p* < 0.0001) greater than that of the vehicle group.

### 3.7. Effect of Chlorich^®^EnergyBoost Supplementation on Serum Lactate Levels Following a 10-Min Swim Test

To evaluate the impact of Chlorich^®^EnergyBoost on blood lactate levels, a 10-min unloaded swimming test was conducted following a 4-week supplementation period. Initial measurements taken before the swimming revealed no significant differences among the groups, as outlined in Table 1. Post-exercise serum lactate levels were notably lower in the supplemented groups compared to the vehicle group, with values recorded at 7.1 ± 0.7 mmol/L for the vehicle, 6.1 ± 0.5 mmol/L for both 1X and 2X, and 5.5 ± 0.2 mmol/L for 5X. The reductions in lactate levels were 14.08% (*p* = 0.0017), 14.08% (*p* = 0.0015), and 22.54% (*p* < 0.0001) for 1X, 2X, and 5X, respectively.

The lactate production rate, calculated as the post-swimming lactate level divided by the pre-swimming level, exhibited reductions of 14.29% (*p* = 0.0014), 11.33% (*p* = 0.0086), and 22.66% (*p* < 0.001) for 1X, 2X, and 5X, respectively, in comparison to the vehicle group. Following a 20-min recovery period, the reductions in serum lactate were further pronounced, with 1X, 2X, and 5X showing decreases of 20.00% (*p* = 0.0001), 27.27% (*p* < 0.0001), and 34.55% (*p* < 0.0001), respectively.

The lactate clearance rate, a metric of recovery efficiency, was computed after 10 min of exercise followed by a 20-min rest period. The clearance rates for 1X, 2X, and 5X were 0.28 ± 0.09, 0.34 ± 0.08, and 0.35 ± 0.06, respectively, in contrast to 0.22 ± 0.14 for the vehicle group. Remarkably, the lactate clearance rates for 2X and 5X were significantly enhanced, being 1.55-fold (*p* = 0.0389) and 1.59-fold (*p* = 0.0389) higher than that of the vehicle group, indicating improved metabolic recovery post-exercise with Chlorich^®^EnergyBoost supplementation.

### 3.8. Effect of Chlorich^®^EnergyBoost Supplementation on Blood Urea Nitrogen Following a 90-Min Swim Test and 60-Min Rest

**Figure 3:**
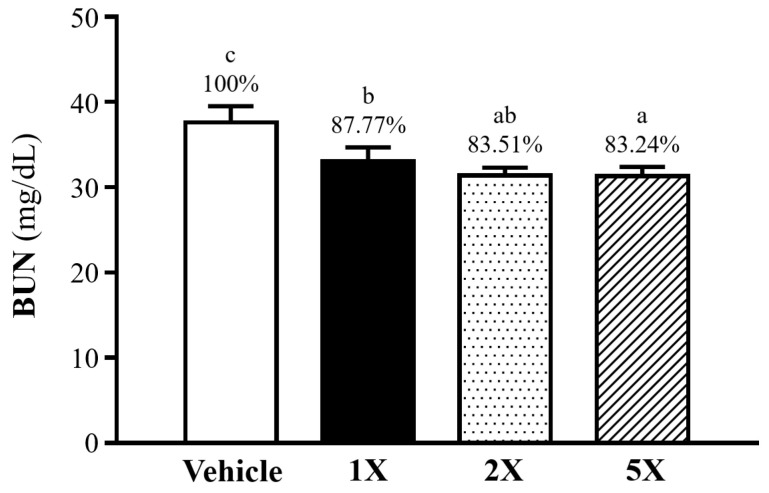
Effect of Chlorich^®^EnergyBoost Supplementation on Blood Urea Nitrogen Levels in Mice. The decrease in BUN levels among the experimental groups: 1X (One fold), 2X (Two fold), and 5X (Five fold), with the vehicle group serving as the control. All results are presented as mean ± SD (*n* = 6 mice per group). Distinct superscript letters (a, b, c) above each data bar indicate statistically significant differences between the groups (*p* < 0.05).

To assess the influence of Chlorich^®^EnergyBoost on metabolic waste elimination, BUN levels were assessed 60 min after a 90-min exhaustive swimming test. The results revealed a significant reduction in BUN levels among the Chlorich^®^EnergyBoost supplemented groups compared to the vehicle group. Specifically, BUN levels were 37.6 ± 1.9 mg/dL in the vehicle group, and 33.0 ± 1.7 mg/dL, 31.4 ± 0.9 mg/dL, and 31.3 ± 1.1 mg/dL in the 1X, 2X, and 5X groups, respectively (refer to Figure 3). The administration of Chlorich^®^EnergyBoost led to reductions in BUN levels by 12.23% in 1X (*p* < 0.001), 16.49% in 2X (*p* < 0.001), and 16.76% in 5X (*p* < 0.0001). These results highlight the dose-dependent effectiveness of Chlorich^®^EnergyBoost in lowering serum BUN levels, indicating enhanced metabolic efficiency and improved clearance of metabolic waste.

### 3.9. Effect of Chlorich^®^EnergyBoost Supplementation on Hepatic and Muscular Glycogen Levels

**Figure 4:**
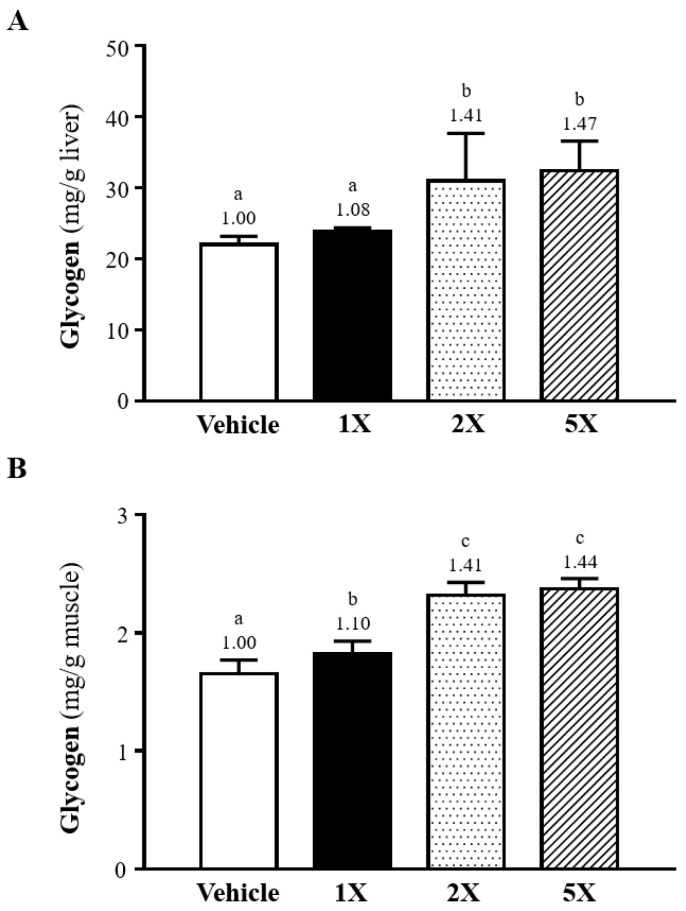
(**A**,**B**) Effects of Chlorich^®^EnergyBoost Supplementation on Muscle and Hepatic Glycogen Levels. Data are reported as mean ± SD (*n* = 6 mice per group). Chlorich^®^EnergyBoost supplementation levels are categorized as 1X (One fold), 2X (Two fold), and 5X (Five fold), with the vehicle group serving as the blank control. The presence of different superscript letters (a, b, c) above each bar indicates statistically significant differences.

To evaluate the impact of Chlorich^®^EnergyBoost on glycogen storage, glycogen levels in both liver and skeletal muscle tissues were quantified after the supplementation period. Figure 4A demonstrates a significant enhancement in glycogen storage within the livers across the supplemented groups. Specifically, hepatic glycogen levels were 22.0 ± 1.2 (mg/g liver) in the control group, and 23.8 ± 0.6, 31.0 ± 6.7, and 32.4 ± 4.2 (mg/g liver) in the 1X, 2X, and 5X groups, respectively. Compared to the control group, the increases in hepatic muscle glycogen levels were 1.41-fold (*p* = 0.0008), and 1.47-fold (*p* = 0.0002) for 2X and 5X, respectively, indicating a significant improvement in glycogen synthesis or conservation. The results, depicted in Figure 4B, exhibit a notable enhancement in glycogen storage within the skeletal muscle across the supplemented groups. Specifically, skeletal muscle glycogen levels were 1.65 ± 0.12 (mg/g muscle) in the control group, and 1.82 ± 0.11, 2.32 ± 0.11, and 2.37 ± 0.09 (mg/g muscle) in the 1X, 2X, and 5X groups, respectively. Compared to the control group, the increases in skeletal muscle glycogen levels were 1.10-fold (*p* = 0.0151), 1.41-fold (*p* < 0.0001), and 1.44-fold (*p* < 0.0001) for 1X, 2X, and 5X, respectively, indicating a dose-dependent enhancement in glycogen synthesis or conservation.

## 4. Discussion

Regular physical activity plays a vital role not only in improving health and fitness but also in enhancing the overall quality of life. Modern challenges, particularly chronic physical fatigue, significantly reduce physical activity levels. Fatigue, characterized by energy depletion, reduced strength, tiredness, and weakness, is widespread and significantly affects daily functioning. Common symptoms of fatigue include tiredness, weakness, and decreased muscle strength [11], impacting both healthy individuals and those with underlying medical conditions. Consequently, there is a growing demand for natural nutraceutical supplements that boost physical energy, alleviate fatigue, and enhance muscle strength [38,39]. Globally, consumers invest billions of dollars in nutritional supplements, prompting a shift towards exploring natural nutrient sources to meet market demands [38]. Among the most popular supplements are branched-chain amino acids (BCAA) and protein supplements [40]. Studies have shown that BCAA and protein supplementation (1–2 g/kg/day) can reduce body protein breakdown and enhance recovery after physical activity [41,42]. However, failure to achieve this may lead to increased fatigue and elevated ammonia levels [18]. This has prompted scientific researchers to seek out natural active substances with the potential to reduce fatigue, promote rapid recovery, and enhance physical strength. Among these, MLP derived from *chlorella* could serve as an alternative nutraceutical component that enhances physical endurance and reduces fatigue [43]. Previous studies have demonstrated that polysaccharides have anti-fatigue properties and can increase physical strength [44,45].

In the current study, hot water extraction combined with an enzymatic approach was utilized to extract *Chlorella sorokiniana* VB-1974, which contains 7% MLP (data not yet published). Twenty-four adult ICR mice were supplemented with Chlorich^®^EnergyBoost for 4 weeks at three different dosages (1-fold, 2-fold, and 5-fold). Serum lactate, BUN, glycogen levels, and swimming performance were analyzed. The results showed that Chlorich^®^EnergyBoost effectively reduced serum lactate levels, improved serum lactate clearance, BUN levels, and muscle glycogen content. The rationale behind this approach is based on the hypothesis that both anaerobic and aerobic exercise performance, as well as physical endurance, require rapid lactate clearance, sufficient muscle glycogen storage, reduced exhaustion time, and minimal muscle wastage. Additionally, genotoxicity tests, acute oral toxicity assessments, and MALDI-TOF MS analyses were conducted to evaluate toxicity and bioactive peptides. The findings revealed no genotoxicity or acute oral toxicity, with MALDI-TOF MS identifying dihydrolipoamide dehydrogenase and SODs peptides present in Chlorich^®^EnergyBoost.

*Chlorella* extract is renowned for its abundant nutrient profile and various nutraceutical benefits, including immune support, antiviral properties, antioxidant capacity, and anti-aging effects, making it a valuable supplement for enhancing well-being [17,20,21]. However, comprehensive data on the effects of *Chlorella sorokiniana* extract, particularly on physical outcomes and the underlying physiological mechanisms, are limited and warrant further investigation.

The findings of this study demonstrate that Chlorich^®^EnergyBoost significantly enhances weight-loaded swimming endurance in mice, indicating improved exercise duration and physical strength. This enhancement is primarily attributed to reduced exhaustion times and efficient metabolic processing during physical activity. Importantly, supplementation is linked to decreased serum lactate levels and enhanced clearance rates following strenuous exercise sessions, underscoring its potent anti-fatigue properties [45,46,47].

In line with previous research [48], elevated lactate levels, a product of anaerobic metabolism, and limited exercise duration are predominantly generated in muscles. The study validates that improved lactate clearance notably boosts endurance, supporting the utilization of Chlorich^®^EnergyBoost to enhance exercise performance. Furthermore, Chlorich^®^EnergyBoost supplementation effectively lowers BUN levels and increases glycogen reserves in both the liver and muscles, indicating improved metabolic well-being and exercise recovery [36,37,49]. Liver and muscle glycogen levels are closely tied to energy supply during physical activity. Intense exercise triggers the activation of the nervous system, leading to the release of hormones such as glucagon, glucocorticoids, adrenaline, and others, which stimulate liver glycogenolysis, elevating blood glucose levels to fuel activity [50,51]. These benefits suggest that the supplement may play a vital role in sustaining energy levels and reducing post-exercise recovery times.

In this study, the bioactive compound in Chlorich^®^EnergyBoost is MLP. The potential mechanisms through which Chlorich^®^EnergyBoost alleviates fatigue include a post-exercise reduction in serum lactate concentration, enhanced lactate clearance, increased glycogen storage in the liver and muscles, and the prevention of muscle loss by inhibiting protein breakdown. The study results align with previous research outcomes. Notably, MALDI-TOF analysis identified the presence of dihydrolipoamide dehydrogenase and SODs. Bioactive peptides play essential roles in cellular signaling, hormonal regulation, and physiological metabolism. Glucose is the primary rapid energy source in the human body, metabolized into pyruvate, which is further processed by pyruvate dehydrogenase to produce acetyl-CoA. Dihydrolipoamide dehydrogenase acts as a key regulator for sensing energy status, initiating pathways in the tricarboxylic acid cycle (TCA cycle) that generate NADH, crucial for ATP production and the cellular energy “currency”. SODs are enzymes catalyzing the conversion of superoxide anion radicals into molecular oxygen and hydrogen peroxide, known for their antioxidant properties that safeguard against cellular damage [50]. SODs have been associated with various health conditions, and their use in personal care products and health supplements as antioxidants and anti-aging agents has been highlighted to counteract free radical damage to the skin and body [52]. The MALDI-TOF MS analysis in this study unveiled the presence of SODs in Chlorich^®^EnergyBoost, consistent with the existing literature on SODs.

A decrease in physical strength is a significant manifestation of fatigue and a sedentary lifestyle, leading to a deterioration in physical function and performance. A sedentary lifestyle has been hypothesized to contribute to the development of chronic conditions such as obesity, hypertension, diabetes, stroke, and other disorders. This study demonstrates that Chlorich^®^EnergyBoost can effectively alleviate fatigue and enhance physical performance through multiple biochemical pathways. This is particularly relevant for both athletes and the general population, who stand to benefit from improved energy management and reduced physical fatigue, potentially assisting in the prevention of conditions like sarcopenia. The findings of this study have implications for enhancing metabolic strength, physical performance, reducing muscle wastage, and alleviating fatigue in both the general public and sports athletes.

The study has certain limitations. Enhancements could involve incorporating biochemistry analyses to gain a more comprehensive understanding of metabolic changes. Furthermore, additional human studies are necessary to definitively determine the effects on humans. Exploring the intricate interactions of active compounds between human studies and the biochemical evaluation of the study could further elucidate the impacts of Chlorich^®^EnergyBoost.

## 5. Conclusions

This study provides compelling evidence supporting the effectiveness of Chlorich^®^EnergyBoost in combating fatigue, enhancing muscle strength, and improving exercise performance over a four-week period. Our findings validate that supplementation with Chlorich^®^EnergyBoost yields various favorable outcomes, including notable reductions in serum lactate levels and BUN, coupled with enhanced lactate clearance rates. Furthermore, the supplement significantly enhances glycogen storage in both liver and muscle tissues and improves forelimb grip strength post-exercise. These results not only demonstrate the potential of Chlorich^®^EnergyBoost as a potent anti-fatigue supplement but also emphasize its role in augmenting overall metabolic efficiency and physical performance. Incorporating this supplement into the dietary regimen of the general public or physically active individuals could substantially enhance their exercise capacity and recovery processes.

## Figures and Tables

**Table 1:** Effect of Chlorich^®^EnergyBoost Supplementation on Serum Lactate Levels After a 10-Min Swimming Test.

Time Point	Vehicle	1X	2X	5X
	Lactate (mmol/L)			
Before swimming (A)	3.5 ± 0.3 ^a^	3.5 ± 0.2 ^a^	3.4 ± 0.3 ^a^	3.5 ± 0.3 ^a^
After swimming (B)	7.1 ± 0.7 ^c^	6.1 ± 0.5 ^b^	6.1 ± 0.5 ^b^	5.5 ± 0.2 ^a^
After a 20 min rest (C)	5.5 ± 0.6 ^c^	4.4 ± 0.3 ^b^	4.0 ± 0.2 ^ab^	3.6 ± 0.3 ^a^
Rate of lactate production and clearance				
Production rate = B/A	2.03 ± 0.11 ^c^	1.74 ± 0.15 ^b^	1.80 ± 0.15 ^b^	1.57 ± 0.14 ^a^
Clearance rate = (B − C)/B	0.22 ± 0.14 ^a^	0.28 ± 0.09 ^ab^	0.34 ± 0.08 ^b^	0.35 ± 0.06 ^b^

Chlorich^®^EnergyBoost supplementation levels: 1X (One fold), 2X (Two fold), 5X (Five fold), and Vehicle (Blank Group). All data are expressed as mean ± SD (*n* = 6 mice per group). The presence of distinct superscript letters (a, b, c) indicates statistically significant differences between the experimental groups (*p* < 0.05) in the same row. The lactate production rate (B/A) represents the lactate level post-swimming (B) divided by pre-swimming levels (A). The clearance rate ((B − C)/B) signifies the difference between lactate levels post-swimming (B) and post-20-min rest (C) divided by post-swimming levels (B).

## Data Availability

The original contributions presented in the study are included in the article/Supplementary Materials, further inquiries can be directed to the corresponding authors.

## Supplementary Materials

The following supporting information can be downloaded at: https://www.mdpi.com/article/10.3390/foods13142232/s1, Table S1. Analysis of the Nutraceutical Composition in Chlorich®EnergyBoost. Table S2. Bacterial Reverse Mutation Assay Results. Table S3. Assessment of Body Weight Gain in Acute Oral Toxicity Test. Table S4. Clinical signs observed in Acute Oral Toxicity Test. Table S5. Gross Necropsy Findings in Acute Oral Toxicity Test. Table S6. Effect of Chlorich®EnergyBoost Supplementation on Body Weight, Water and Dietary intake. Figure S1. Animal test timeline. Twenty-four Institute Cancer Research (ICR) mice were were assigned to four groups (mice = 6 in each group). Supplementation levels for the Chlorich®EnergyBoost groups are denoted as 1X (One fold), 2X (Two fold), and 5X (Five fold), with the vehicle group serving as the control. Mice were housed in the respective room for two weeks before the start of the experiment. After four-weeks of Chlorich®EnergyBoost supplementation, grip strength test was performed at 29th day, endurance test was performed at 31st day, lactate tese was performed at 33rd day, blood urea nitrogen (BUN) test was performed at 35th day, glycogen assay was performed at 37th day after start of the supplementation. Weight changes were recorded through out the experiment..
