# Supplementary material for: Effects of Chlorich®EnergyBoost on Enhancing Physical Performance and Anti-Fatigue Properties in Mice
Source: Foods. 2024 Jul 16;13(14):2232. doi: 10.3390/foods13142232 (PMC11275582; doi:10.3390/foods13142232)
Supplement: Supplementary file 1 [file foods-13-02232-s001.zip › foods-3080935-supplementary.pdf]

**Table S1.** Analysis of the Nutraceutical Composition in Chlorich®EnergyBoost

| **Nutrients** | Main components |
|---|---|
| Carbohydrate | Polysaccarides, $\alpha$-1,4-glucose, $\beta$-1,6 glucose |
| Protein | 18 types of amino acids (include EAA and BCAA) |
| Lipids | Polysaturated fatty acids and ($\alpha$-linolenic aicd, ;omp;eoc acid),<br>Monosaturated fatty acids (Oleic acid) |
| Minerals | Potassium, Calcium, Iron, Zinc, Magnesium, Phosphorus |

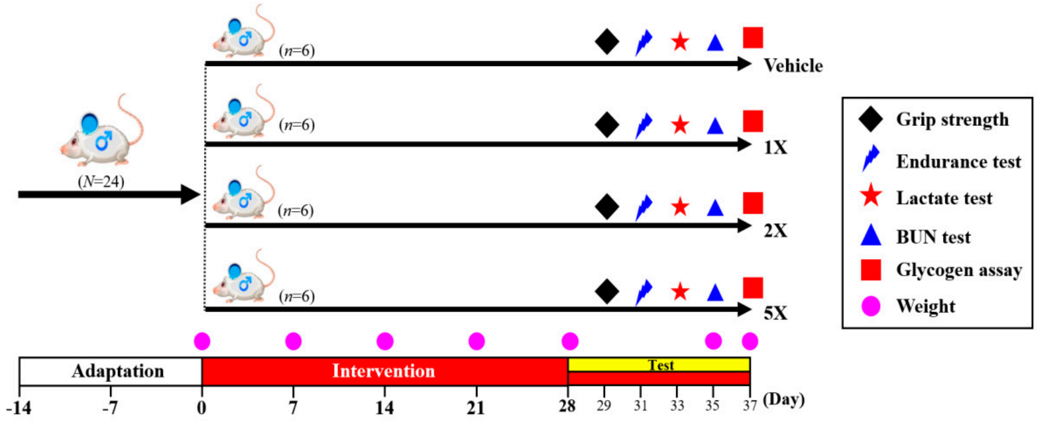


**Figure S1**. Animal test timeline. Twenty-four Institute Cancer Research (ICR) mice were were assigned to four groups (mice = 6 in each group). Supplementation levels for the Chlorich®EnergyBoost groups are denoted as 1X (One fold), 2X (Two fold), and 5X (Five fold), with the vehicle group serving as the control. Mice were housed in the respective room for two weeks before the start of the experiment. After four-weeks of Chlorich®EnergyBoost supplementation, grip strength test was performed at 29th day, endurance test was performed at 31st day, lactate tese was performed at 33rd day, blood urea nitrogen (BUN) test was performed at 35th day, glycogen assay was performed at 37th day after start of the supplementation. Weight changes were recorded through out the experiment.

**Table S2.** Bacterial Reverse Mutation Assay Results

| Group (mg/plate) | | Number of revertants/plate (without S9 activator, Mean±SD, *n*=3) | | | | |
|---|---|---|---|---|---|---|
| | | **TA98** | **TA100** | **TA102** | **TA1535** | **TA1537** |
| Negative control | | 33.0 ± 3.0 | 115.0 ± 16.4 | 351.3 ± 10.1 | 23.0 ± 2.6 | 12.7 ± 1.2 |
| Aqueous extract of *Chlorella sorokiniana* | 0.05 | 37.7 ± 0.6 | 116.3 ± 15.3 | 324.0 ± 20.9 | 22.7 ± 3.2 | 12.7 ± 1.5 |
| | 0.16 | 41.7 ± 4.0 | 128.7 ± 3.8 | 326.7 ± 4.6 | 32.3 ± 2.1 | 10.0 ± 2.0 |
| | 0.50 | 47.7 ± 2.3 | 131.0 ± 10.1 | 321.3 ± 10.1 | 29.0 ± 1.0 | 14.0 ± 1.0 |
| | 1.58 | 39.0 ± 3.0 | 127.3 ± 5.7 | 332.7 ± 16.8 | 29.7 ± 1.2 | 18.7 ± 2.1 |
| | 5 | 39.0 ± 5.0 | 136.3 ± 15.0 | 332.0 ± 14.4 | 34.3 ± 3.8 | 10.7 ± 1.2 |
| Positive control | | 204.7 ± 23.4* | 563.3 ± 39.3* | 2221.3 ± 84.8* | 484.7 ± 33.5* | 725.0 ± 58.0*# |
| Group (mg/plate) | | Number of revertants/plate (with S9 activator, Mean±SD, *n*=3) | | | | |
| Negative control | | 49.3 ± 3.1 | 96.3 ± 6.7 | 354.0 ± 6.9 | 19.3 ± 1.5 | 11.0 ± 3.0 |
| Aqueous extract of *Chlorella sorokiniana* | 0.05 | 52.3 ± 4.5 | 92.0 ± 4.6 | 366.7 ± 12.9 | 15.7 ± 2.1 | 9.7 ± 3.1 |
| | 0.16 | 50.7 ± 2.9 | 94.3 ± 3.1 | 344.0 ± 12.0 | 13.0 ± 2.6 | 11.0 ± 1.7 |
| | 0.50 | 47.7 ± 3.5 | 93.0 ± 4.4 | 342.7 ± 15.1 | 15.0 ± 1.7 | 10.0 ± 2.0 |
| | 1.58 | 56.7 ± 3.2 | 100.7 ± 11.6 | 386.7 ± 16.2 | 17.0 ± 3.5 | 9.0 ± 2.6 |
| | 5 | 55.0 ± 4.6 | 113.3 ± 4.7 | 322.7 ± 9.9 | 17.7 ± 2.1 | 12.3 ± 3.8 |
| **Positive control** | | 466.0 ± 17.8* | 310.0 ± 15.9* | 914.7 ± 102.6* | 172.0 ± 25.0* | 439.3 ± 15.3* |

**Table S3.** Assessment of Body Weight Gain in Acute Oral Toxicity Test

| Study Duration | Mean Body Weight Gains (g) | |
|---|---|---|
| | Group 1 (10000 mg/kg) Male | Group 1 (10000 mg/kg) Female |
| Day 1–8 | 70.08±5.23 | 30.78±2.85 |
| Day 8–15 | 52.14±5.89 | 16.96±7.80 |
| Total Gains (Day 1–15) | 122.22±8.09 | 47.74±8.70 |

All data expressed as mean±SD (*n*=10 for both sexes of Sprague Dawley rates per group).

**Table S4.** Clinical signs observed in Acute Oral Toxicity Test

| Group | Clinical Signs | Incidence (N/N) [a] | | Total Incidence (n′/n′) [b] |
|---|---|---|---|---|
| | | Day 1-7 | Day 8-15 | |
| 1 (10000 mg/kg) Male | Abnormality | 0/10 | 0/10 | 0/10 |
| 1 (10000 mg/kg) Female | | 0/10 | 0/10 | 0/10 |

**Table S5.** Gross Necropsy Findings in Acute Oral Toxicity Test

| Group | Sex | Abnormal Gross Necropsy Findings | Total Incidence (N/N)[1] |
|---|---|---|---|
| 1 (10000 mg/kg) | Male | No abnormal findings | 0/10 |
| 1 (10000 mg/kg) | Female | No abnormal findings | 0/10 |

**Table S6.** Effect of Chlorich®EnergyBoost Supplementation on Body Weight, Water and Dietary intake.

| Characteristics | Vehicle | 1X | 2X | 5X |
|---|---|---|---|---|
| Initial BW (g) | 32.3±1.0 [a] | 32.2±0.5 [a] | 32.4±1.4 [a] | 32.4±1.0 [a] |
| 1st wk BW (g) | 33.5±1.1 [a] | 33.9±0.9 [a] | 33.4±1.7 [a] | 33.7±1.1 [a] |
| 2nd wk BW (g) | 34.5±1.4 [a] | 34.8±0.9 [a] | 34.4±1.7 [a] | 34.4±2.7 [a] |
| 3rd wk BW (g) | 35.8±1.6 [a] | 35.9±0.7 [a] | 35.6±1.7 [a] | 35.7±2.7 [a] |
| 4th wk BW (g) | 36.4±1.6 [a] | 36.7±0.7 [a] | 36.5±1.3 [a] | 36.9±2.8 [a] |
| Final BW (g) | 36.6±1.5 [a] | 36.9±1.0 [a] | 36.9±1.6 [a] | 37.3±2.5 [a] |
| Water intake (mL/mouse/day) | 6.2±0.9 [a] | 6.2±0.9 [a] | 6.2±0.9 [a] | 6.1±1.0 [a] |
| Diet (g/mouse/day) | 6.3±0.9 [a] | 6.1±1.1 [a] | 6.1±1.1 [a] | 6.1±1.0 [a] |

Supplementation levels for the Chlorich®EnergyBoost groups are denoted as 1X (One fold), 2X (Two fold), and 5X (Five fold), with the vehicle group serving as the control. Data is presented as mean±SD (*n*=6 mice per group). The superscript "a" indicates no statistically significant differences within the same result row.
